# Cancer and non-cancer brain and eye effects of chronic low-dose ionizing radiation exposure

**DOI:** 10.1186/1471-2407-12-157

**Published:** 2012-04-27

**Authors:** Eugenio Picano, Eliseo Vano, Luciano Domenici, Matteo Bottai, Isabelle Thierry-Chef

**Affiliations:** 1 Institute Clinical Physiology of the National Research Council CNR, 56124, Pisa, Italy; 2 San Carlos University Hospital, Complutense University, Medical Physics Service, Madrid, Spain; 3 Neuroscience Institute of the National Research Council, CNR, Pisa, and Scienze e Tecnologie Biomediche Department, L'Aquila University, Pisa, Italy; 4 Institute of Environmental Medicine, Karolinska Institutet, Karolinska, Sweden; 5 Environment and Radiation, International Agency for Research on Cancer, Lyon, France

**Keywords:** Brain cancer, Cognitive effects, Interventional cardiologist, Radiation exposure, Risk

## Abstract

****Background**:**

According to a fundamental law of radiobiology (“Law of Bergonié and Tribondeau”, 1906), the brain is a paradigm of a highly differentiated organ with low mitotic activity, and is thus radio-resistant. This assumption has been challenged by recent evidence discussed in the present review.

****Results**:**

Ionizing radiation is an established environmental cause of brain cancer. Although direct evidence is lacking in contemporary fluoroscopy due to obvious sample size limitation, limited follow-up time and lack of focused research, anecdotal reports of clusters have appeared in the literature, raising the suspicion that brain cancer may be a professional disease of interventional cardiologists. In addition, although terminally differentiated neurons have reduced or mild proliferative capacity, and are therefore not regarded as critical radiation targets, adult neurogenesis occurs in the dentate gyrus of the hippocampus and the olfactory bulb, and is important for mood, learning/memory and normal olfactory function, whose impairment is a recognized early biomarker of neurodegenerative diseases. The head doses involved in radiotherapy are high, usually above 2 Sv, whereas the low-dose range of professional exposure typically involves lifetime cumulative whole-body exposure in the low-dose range of < 200 mSv, but with head exposure which may (in absence of protection) arrive at a head equivalent dose of 1 to 3 Sv after a professional lifetime (corresponding to a brain equivalent dose around 500 mSv).

****Conclusions**:**

At this point, a systematic assessment of brain (cancer and non-cancer) effects of chronic low-dose radiation exposure in interventional cardiologists and staff is needed.

## Review

The characterization of health effects (cancer and non-cancer) of chronic low-dose radiation (LDR) is still incomplete and difficult. The UNSCEAR (United Nations Scientific Committee on the Effects of Atomic Radiation) 2009 clearly recommends paying more attention “to other non-cancer disease entities, in addition to circulatory diseases”, encouraging “future epidemiological studies designed to assess clinical and subclinical endpoints, as well as biomarkers, since this information is more likely to lead to insights” [1]. In 2006 the National Academy of Sciences BEIR VII committee identified as one of the top ten research needs “future occupational radiation studies”, which should include highly exposed populations with full record of exposure and well-suited to assessing the effects of long-term, low-level radiation exposure in humans [2]. The International Commission on Radiological Protection (ICRP) stated in 2011 that “particular attention should be paid to radiation effects in the lens of the eye and on the cardiovascular system, because of recent published observations of radiation effects in these systems occurring at much lower doses than reported previously”, and that brain irradiation can have direct radiation effects on the thyroid and pituitary glands, as well as subtle effects on the hypothalamic-pituitary-adrenal axis and the hypothalamic, pituitary-gonadal axis [3].

Within the general framework of the still-elusive assessment of cancer and non-cancer effects of LDR, high and unprecedented levels of radiation exposure in the contemporary population of interventional cardiologists and other paramedical staff working at the catheterization laboratory clearly represent a challenge and an opportunity, especially if we wish to characterize the brain effects of LDR. The brain is a paradigm of a highly differentiated organ with low mitotic activity, thus considered radio-resistant according to a fundamental law of radiobiology (“law of Bergonié and Tribondeau”, 1906). In fact, the brain is one of the main target organs of radiation exposure in the catheterization lab [4,5], and is usually unprotected due to the myth of its radio-resistance [6]. The brain and head effects of LDR may include stochastic and deterministic effects. Stochastic or probabilistic effects of low-dose radiation consist primarily of cancer, which is the main effect recognized at a regulatory and radioprotection level [7]. In theory, stochastic effects may well include other non-cancer effects such as neuro-vascular and neuro-degenerative effects, for which there is clear experimental evidence [8]. Another clinically relevant radiation effect on the head is eye cataract, previously thought to be deterministic (tissue reactions) and currently recognized as possibly stochastic in nature, and occurring at much lower radiation exposure level than previously thought [9]. In general, there is a striking lack of evidence systematically collected in exposed medical professionals.

### Radiation exposure of interventional cardiologists

**Figure 1 (a):**
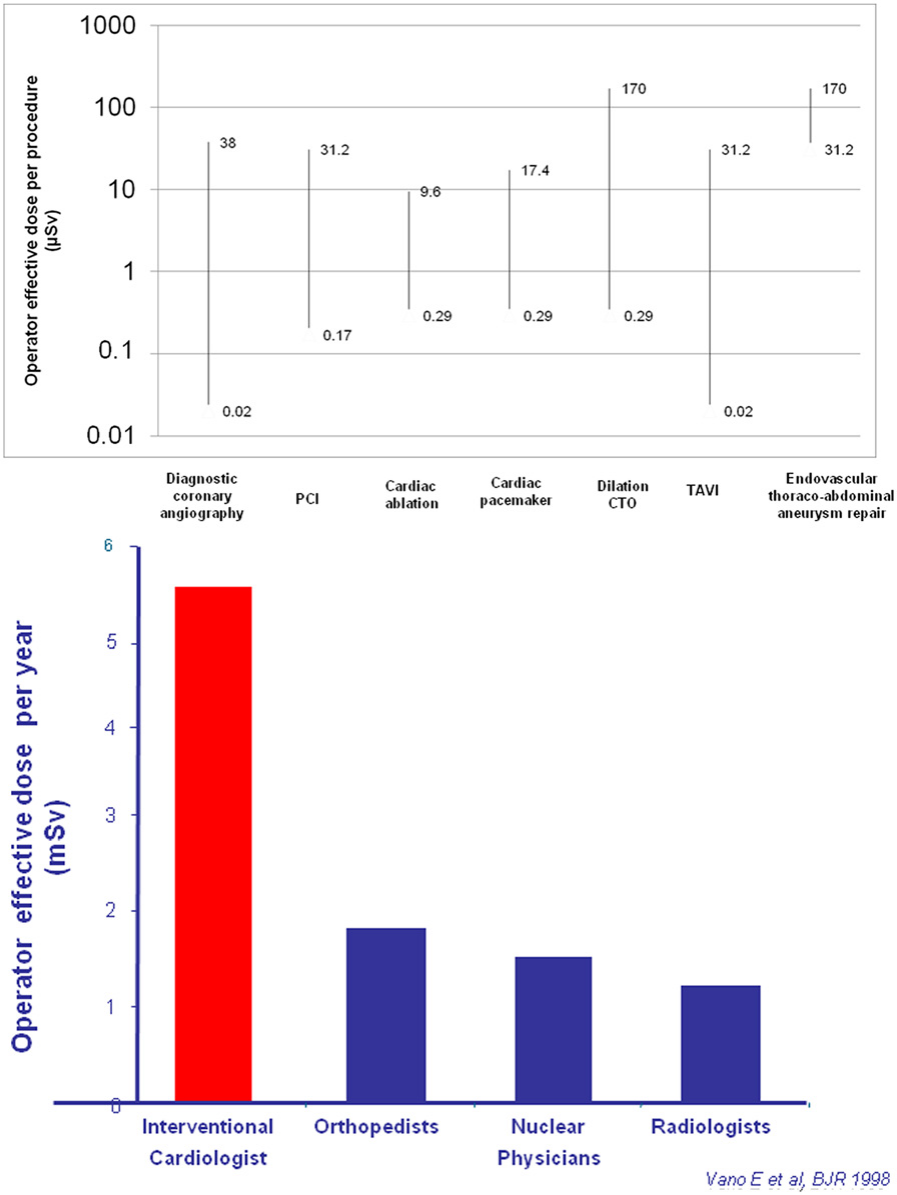
** The range of radiation exposure of interventional cardiologists per single procedure: diagnostic coronary angiography, percutaneous coronary angioplasty, cardiac ablation, cardiac pacemakers, intracardiac defibrillator or implantation, transcutaneous aortic valve implantation, dilation of chronic coronary total occlusion and endovascular thoraco-abdominal aneurysm repair.** From original data of references 14–16. There is substantial variability in operator dose across procedures and within each procedure. On the y-axis, a log scale is used. Right side: The annual radiation exposure for different specialists. Interventional cardiologists are by far the most exposed (modified from Vano E et?al., 1998, ref. 4). **(b)** The map of radiation exposure in the cardiac interventionalist. There are “hot regions” of higher exposure in the eye, thyroid and brain that should be carefully protected by glasses, collars and cap. Radiation exposure on the left is almost double that on the right side (modified from Vano E et?al., 1998, ref. 4). Right side: Estimated cumulative dose after 20 years of professional life in the cardiac cath lab: the whole body dose (below lead apron) is around 100 mSv; the head dose is 10 times higher, and in the head, the left head dose is twice that of the right side dose. Obviously, in this order of magnitude there are substantial variations (up to 10 times) depending on years of exposure, volume of activity, type of procedure, technology used, protection habits and radiation awareness.

**Figure 2:**
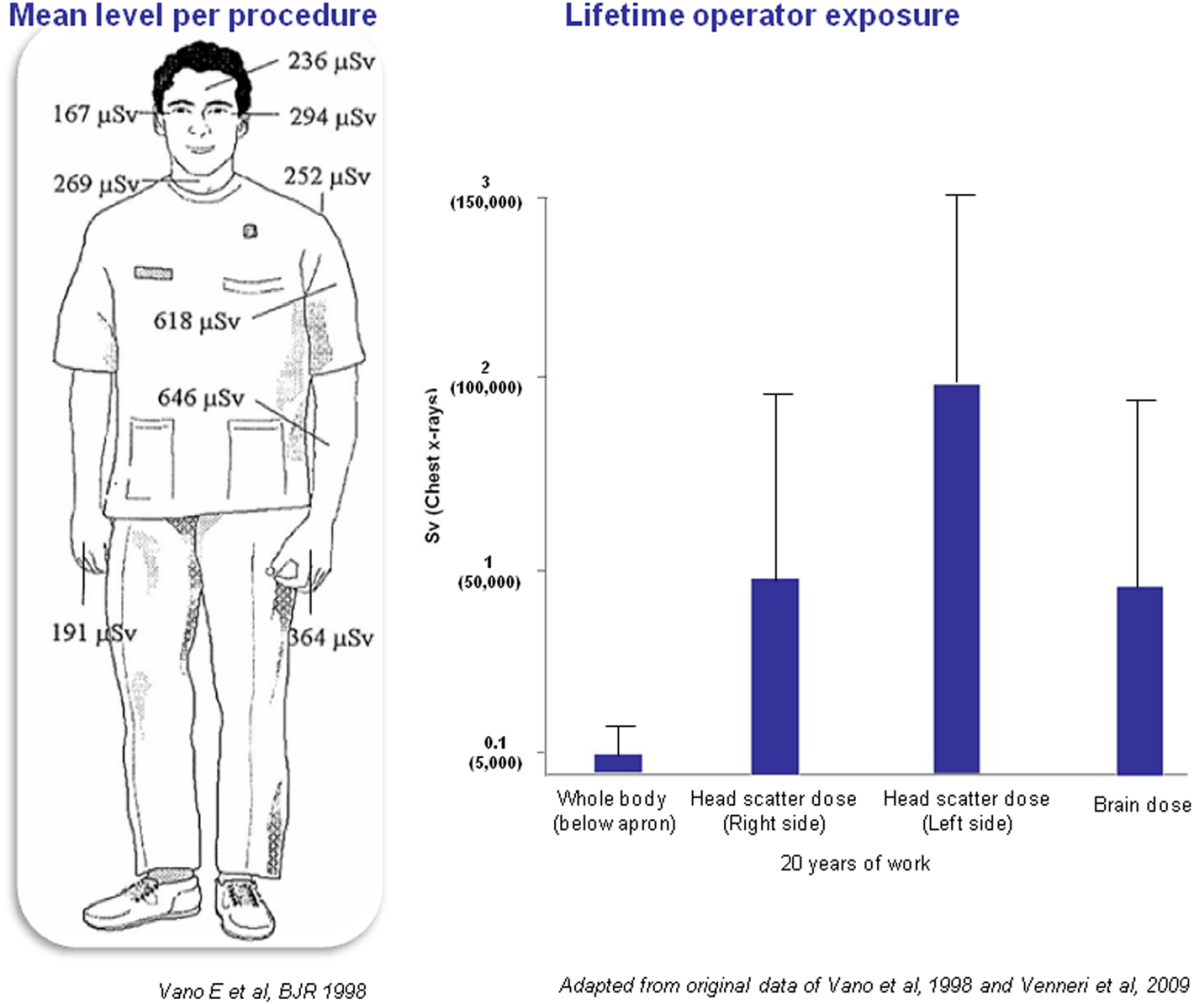
**The dose-effect relationship between radiation exposure and cancer (left side) and radiation exposure and atherosclerosis (right side).** The solid line indicates the epidemiological evidence, which is conclusive for cancer risk above 50 mSv and for atherosclerosis risk above 500 mSv. The dashed line indicates the dose range with absent or inconclusive evidence. Freely adapted from refs 3 (BEIR VII, 2006) and 7 (ICRP publication 103, 2007).

Medical radiation from x-rays and nuclear medicine is the largest manmade source of radiation exposure in Western countries, accounting for a mean effective dose of 3.0 mSv per capita per year, corresponding to a cardiological risk of 150 chest x-rays [10,11]. Of this equivalent 150 chest x-rays from medical radiation (except radiotherapy), almost one-half come from cardiology procedures [11]. Interventional radiology and interventional cardiology account for about 14% (0.43 of 3.0 mSv) of overall exposure to the average US citizen for the radiological year 2006 [11]. Each procedure involves a relatively large radiation exposure for the patient, which in each exam may range from 7 to 56 mSv, around an average reference dose of 15 mSv for a percutaneous coronary intervention or a cardiac radiofrequency ablation [12]. The high levels of patient exposure also imply a significant professional exposure for the interventional cardiologist, who needs to operate near the patient and the radiation source. The single dose per procedure of the operator is on the order of magnitude of one thousandth of the exposure of the patient [13] (Figure 1a). Effective occupational doses per procedure range from 0.02 to 38 microSv for diagnostic catheterization, 0.2 to 31.2 microSv for percutaneous coronary intervention, 0.2–9.6 microSv for ablation, 0.3–17.4 microSv for pacemaker or intracardiac defibrillation implantations [14] and may reach even higher values per procedure up to 50 microSv for dilation of chronic total occlusion and up to 100 microSv transcutaneous aortic valve [15] and up to 200 microSv per single procedure of endovascular thoraco-abdominal aneurysm repair [16]. The measurement over personal protective devices ranged from 0.4–1,100 microSv at the eye level, 1.2–580 microSv at the thyroid level, 32–750 microSv at the trunk level, and 0.4–790 microSv at head level, whereas measurements under the apron at the trunk levels ranged from 0 to 23 microSv [14]. Each operator does hundreds or thousands of procedures each year, and therefore the cumulative dose in a professional lifetime is not negligible. The most active and experienced interventional cardiologists in high-volume cath labs have an annual exposure equivalent to around 5 mSv (below apron) per year, two to three times higher than that of diagnostic radiologists [4] (Figure 1b) and a projected professional lifetime attributable excess cancer risk of 1 in 100 [17]. Of special concern, in interventional cardiologists the head organ dose is 10- to 20-fold higher than the dose recorded beneath the apron [18,19] (Figure 2). Annual exposure to the cardiologist's head is on the order of magnitude of 100 microsieverts per single ablation procedure [18] and in the range of 20–30 mSv per year [19] or much higher if a ceiling-suspended screen is not used [20,21]. Radiation from the fluoroscopy tube is scattered by the patient while the cardiac intervention is underway and can reach the physician’s head, which is often unprotected, even if a lead apron is worn to protect the torso. The left side of the operator is more exposed than the right side in most cases due to the usual layout of an interventional room, where the radiologist or cardiologist operates from the right side of the patient. Scattered radiation comes from the patient and is more intense when the x-ray tube is on his/her left. This implies that the lifetime estimated organ dose for a busy interventional cardiologist after 25 years of work in the catheterization laboratory is in the order of magnitude of 1 to 3 Sv as scatter dose measured at the head with a dosimeter and the brain dose that could approximately be a 20–25% of the “external dose” (Figure 2). Unfortunately, the practice of interventional cardiology is sometimes accompanied by suboptimal perception of radiation risk and by negligent use of radiation protection tools [22-24]. Radioprotection awareness by operators is dramatically effective in reducing professional exposure by 90% [20]. Today, in most cardiology imaging laboratories and in interventional radiology fluoroscopy rooms, overhead radiation shields, thyroid shields, and leaded aprons are employed to reduce the radiation doses to the operators head and neck. It is rare that unprotected radiologists or cardiologist would do an angiography procedure. Unfortunately, this was not the most common situation in the past, and still today is not the rule in all and every laboratory [22-24].

### Brain cancer risk

The central dogma of radioprotection is that biological effects of ionizing radiation are a direct consequence of DNA damage occurring in directly irradiated cells. This remains a useful approach in radioprotection, although in experimental models of mice medulloblastoma, brain cancer increases in spite of lead shields for protecting mouse heads due to a oncogenic “bystander radiation effect”, probably linked to soluble factors released by irradiated cells [25]. In addition to the absorbed dose and type of radiation, the probability of stochastic effects varies depending on the organ or tissue irradiated. To calculate the effective dose, the individual organ dose values (equivalent doses) are multiplied by the respective dimensionless tissue weighing factor. In radiation protection, the tissue weighting factor is a factor weighting the equivalent dose in a particular tissue or organ in terms of its relative contribution to the total deleterious effects resulting from uniform irradiation of the whole body. In other words, the higher this factor, the more radiosensitive the tissue. The ICRP has offered recommended tissue weighting factors in three reports, their Publication 26 (1977) [26], Publication 60 (1991) and the most recent Publication 103 (2007) [7]. The key changes introduced in ICRP Publication 103 are a 140% increase in breast risk factor from 0.05 to 0.12 (a value similar to colon, lung, stomach and red bone marrow, all considered highly radiosensitive tissues) and a decrease in gonad weighting from 0.20 to 0.08 (Table 1). In 2007, brain tissue was given more weight and received 0.01 of weighting factor, whereas it was clustered among the remainder (with 14 other tissues) in the 1991 version. A similar trend was shown for the salivary glands, which may also suffer from high head irradiation in catheterization laboratory workers.

**Table 1:** Tissue weighting factors from ICRP (2007 vs 1991 and 1997)

	**ICRP 26**^**8**^**(1977)**	**ICRP 60 (1991)**	**ICRP 103**^**7**^**(2007**
Bladder	_ _ _	0.05	0.04
Bone	0.03	0.01	0.01
Brain	_ _ _	_ _ _	0.01
Breasts	0.15	0.05	0.12
Colon	_ _ _	_ _ _	0.12
Esophagus	_ _ _	0.05	0.04
Liver	_ _ _	0.05	0.04
Lower large intestine	_ _ _	0.12	_ _ _
Lungs	0.12	0.12	0.12
Ovaries/testes	0.25	0.20	0.08
Red marrow	0.12	0.12	0.12
Remainder tissues	0.30	0.05	0.12
Salivary glands	_ _ _	_ _ _	0.01
Skin	_ _ _	0.01	0.01
Stomach	_ _ _	0.12	0.12
Thyroid		0.05	0.04

Adapted from refs 7, 26 and 27.

Shifting from a radioprotection to an oncology perspective, ionizing radiation is one of the few established causes of neural tumors. The sensitivity of the brain tissues to develop benign and malignant tumors after diagnostic X-rays was shown in several case–control studies, four of them from dental exposures, and with relative risks ranging from 1.6 to 10 [27]. Studies of the incidence of nervous system tumors in atomic bomb survivors concluded that exposure to radiation doses of less than 1 Sv is associated with an increased incidence of nervous system tumors [28]. A review of cohort mortality studies among workers exposed to ionizing radiation in U.S. nuclear programs was reported in 1991 and reappraised in 2001 [29], with 3.8 person-years of observation among 140,000 white male workers. The increased risk of brain tumor was highly consistent, persistent, and stable, on the order of magnitude of 15–30%. As a consequence of these data, policy makers have identified brain cancer as a "specified" cancer potentially related to occupational exposures under the Energy Employees Occupational Illness Compensation Program Act [30]. Cosmic radiation can also probably provoke brain tumors. In a large German cohort of 6,017 cockpit and 20,757 cabin crew members, Zeeb et al. reported an increased fatal brain tumor risk among cockpit (not cabin) crew, with the relative risks of 1, 2.49 and 3.56 for workers with 10–20 years, 20–30 years, and > 30 years duration of employment, respectively [31].

Epidemiologic evidence for radiation-induced brain cancer in fluoroscopists is suggestive, but by no means conclusive (Table 2) [32-40]. One study [32] found that the death rate from brain cancer in radiologists was almost three times that of other medical specialists who did not use radiation. A case–control study [37] of 233 patients with brain tumors reported that work as a physician with use of fluoroscopy increased the risk of developing a brain tumor, with an odds ratio of 6.0 (95% CI, 0.62–57.7), although there were only three such individuals among the 233 cases. Another case–control study [35] of 476 individuals diagnosed with glioma also observed an increased risk in physicians and surgeons (odds ratio, 3.5; 95% CI, 0.7–17.6). However, such studies cannot exclude other biologic agents and chemicals unrelated to radiation as causative, and other case–control studies failed to identify a significant risk of brain tumors as a result of a generic exposure to medical ionizing radiation [38]. Essentially, no data are available on the contemporary population of invasive fluoroscopists, whose level and pattern of head exposure is unprecedented, although some anecdotal clusters of brain cancer have been recently described [39,40]. Of particular interest, the most recent description of three brain gliomas and one meningioma in interventional cardiologists all involved the left side, known to be more exposed than the right side, although no brain radiation dose was provided for any of these cases [40].

**Table 2:** Reports of brain cancer incidence in physicians, radiologists and interventionalists

**STUDY, YEAR**	**METHODS**	**FINDINGS**
Matanoski et al., 1975 [32]	Cohort study of mortality in 6,500 US male radiologists (years first worked 1920–1969) over a 50-year period	Excess cancer risk among radiologists compared with other physicians
Wang JX et al., 1990 [33]	Cohort study of Chinese diagnostic x-ray workers (1950 to 1985)	Trend of excess cancer risk (standardized incidence ratio 1.2 for employment duration 10–14 years; 2.3 for 15–19 years) compared to non-radiation medical workers, not available for brain cancer
Andersson M et al., 1991 [34]	Cohort study of Danish radiation therapy workers	Trend of excess cancer risk (standardized incidence ratio 1.09 with measured radiation dose < 5 mSv, and 2.23 with dose 5–50 mSv), not available for brain cancer
Carozza et al., 2000 [35]	Case–control study of occupation and glioma	Physicians at increased, albeit imprecise, risk of glioma (OR 3.5, CI 0.7- 17)
Andersen M et al., 1999 [36]	Population-based study of occupation and cancer incidence (from the 1990s to 1980s)	Brain cancer increased among physicians in general; no breakdown by specialty
Hardell et al., 2001 [37]	Case control study of 233 gliomas	Excess cancer risk of 6.0 in fluoroscopists
Blettner et al., 2007 [38]	Case control study of German patients (age 30–59 years at diagnosis) with brain cancer in 2001–2003	Occupational exposure (physicians, nurses, radiographers) with OR 2.49 (0.74–8.38) for neurinoma, OR close to 1 for glioma and meningioma
Finkelstein et al., 1998 [39]	Report of a case cluster (1990s)	Brain cancer in two interventionalists
Roguin et al., 2012 [40]	Report of a case cluster (2000s)	3 brain gliomas and 1 meningioma, left-sided, in 4 interventional cardiologists

In general, we should consider some important methodological aspects: 1) timing of studies; 2) sample size; 3) changing levels of exposure [41,42]. Most studies were conducted at a time when interventional cardiology was still a relatively new phenomenon with low levels of use compared with today. For most known carcinogens, identification of increased risk of solid tumors (particularly brain tumors) has required long follow-up periods of subjects with substantial exposure. For example, while the atomic bombs were dropped on Hiroshima and Nagasaki in August 1945, an excess risk of solid tumors was reported in the survivors only in the 1960s, and no elevation in risk of brain tumors was noted for about 50 years [28]. Another important issue is that exposure from interventional cardiology is very asymmetrical, with the left side twice more exposed that the right side [4,13]. The risk, if it exists, is therefore likely to be more pronounced on the left side. Therefore, studies now at the starting blocks should enroll a well-characterized population of catheterization lab workers, record all (including non-fatal and non-malignant) cases of brain cancer and other non-brain head cancers, such as salivary glands, and assess the possible asymmetry of incidence (left- side cases being possibly more frequent than right-sided cases) mirroring the asymmetry of dose exposure.

Another potentially useful approach is the use of registry data. In some countries of Europe and the US, there are updated and reliable registries that can link occupational exposure with death and hospitalization records for a large number of individuals exposed many years ago [36,38].

### Radiation exposure and vascular disease

**Figure 3:**
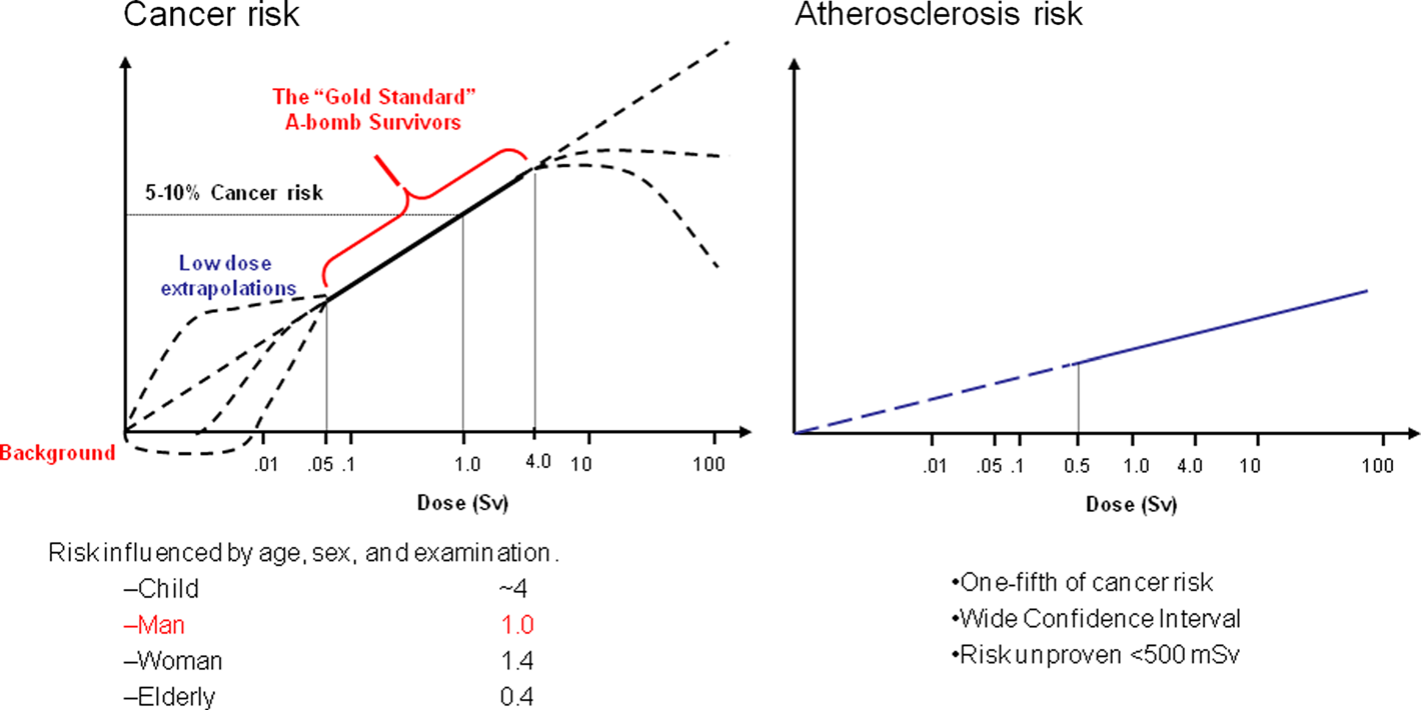
**The modified cellular and pathophysiological model leading to neurodegenerative and atherosclerotic disease through possibly shared molecular pathways.** Modified from ref. 48.

Systematic reviews of the published epidemiological literature and cardiovascular disease [43,44], or reviews of studies of populations medically, occupationally or environmentally exposed to relatively low-dose radiation [45-47] concluded that there is a significant association (although with substantial heterogeneity) between radiation exposure and circulatory disease, either cardiovascular or cerebrovascular. Vascular injury is a well-recognized cause of late radiation-therapy morbidity and this manifests as atherosclerosis in large vessels [7,8]. There is a significant increase in localized atherosclerosis after radiotherapy for head and neck cancer and this results in an excess risk of cardiovascular disease and stroke, which is increasingly evident with long follow-up (> 10 years) [43]. From current evidence, according to ICRP 2011 a judgement can be made regarding a threshold acute dose of about 0.5 Gy (or 500 mSv) for both cardiovascular disease and cerebrovascular disease [3] (Figure 3). On that basis, “0.5 Gy may lead to approximately 1% of exposed individuals developing the disease in question, more than 10 years after exposure. This is in addition to the high natural incidence rate (circulatory disease accounts for 30–50% of all deaths in most developed countries)." [3]. This dose threshold can be reached by an interventional cardiologist in his/her professional life as head dose and by a patient during some complex interventional procedures [3]. Several biological mechanisms exist which might be responsible for linking radiation exposure to atherosclerosis. A relatively recent paradigm states that atherosclerosis is an inflammatory disease of the arteries that can lead to ischemia of the heart and brain, resulting in infarction. Chronic inflammation of the artery is initiated by endothelial dysfunction that may be caused, for example, by elevated low-density lipoproteins, and free radicals. The chronic inflammation results in altered function of macrophages and lymphocytes, whose activation causes the release of cytokines and growth factors leading to increased damage, genomic instability and cell aging, cell apoptosis, local necrosis, and altered blood flow [48] (Figure 3). Clinical laboratory data from the clinical Adult Health Study subset also provide some insight into subclinical changes underlying disease development, such as an increased amount of aortic arch calcification [49], dose-dependent increases in longitudinal trends for systolic and diastolic blood pressure [50], serum cholesterol levels [51], and dose-related increase in serum levels of various inflammation markers among the cohort subjects, including C-reactive protein, interleukin-6 and sialic acid [52], with decreases in proportion of CD4+ T-cells in the peripheral blood lymphocytes, suggesting a role of radiation-induced immunity in promotion of pre-clinical inflammation [53].

**Figure 4:**
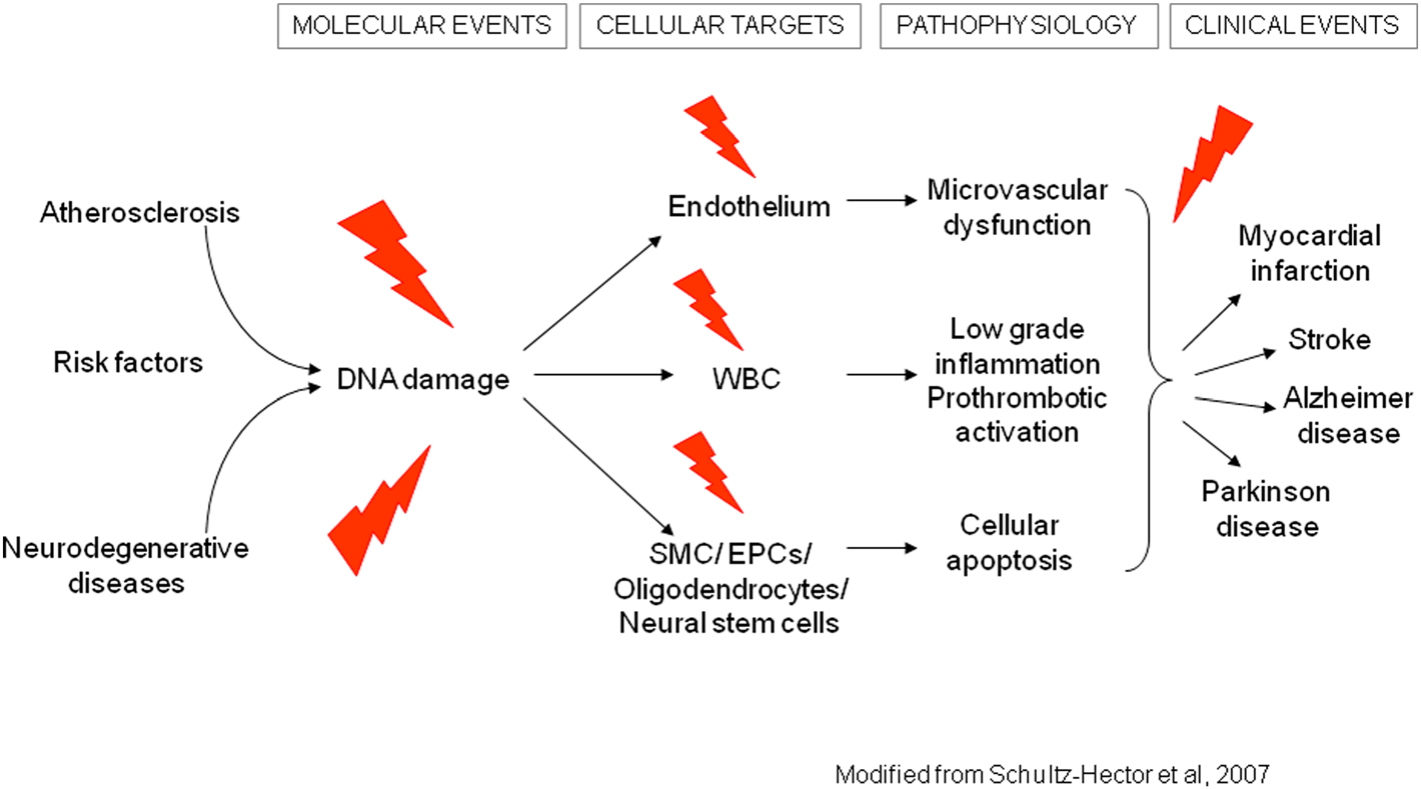
**The cellular and experimental effects of x-ray on adult brain.** Adult neurogenesis occurs in the caudate nucleus, hippocampus and olfactory bulb (left upper panel, A); environmental factors can positively (environmental enrichment) or negatively modulate adult brain plasticity (right upper panel, B); of many physical, chemical and genetic factors modulating plasticity, x-rays are a recognized potent inhibitor of neurogenesis (left lower panel, C). The inhibition of neurogenesis in a mouse model is more striking in males than in females, and with repetitive, chronic rather than with acute exposures (right lower panel, D, modified from ref 70, Silasi et al.).

Radiation effects on the vascular system are not limited to macrovessels such as carotid arteries but may also involve small arterioles and microcirculatory function in vessels too small to be imaged by angiography [54,55]. It is now well-recognized that many of the same risk factors that cause heart disease also can lead to vascular dementia in the elderly [56] and microvascular brain damage – the result of age-associated alteration in large arteries and the progressive mismatch of their cross-talk with small cerebral arteries – a potent risk factor for cognitive decline and the onset of dementia in older individuals [57]. Morphological and functional alterations of the dermal microcirculation identified by capillary microscopy have been identified in 145 physicians exposed to low-dose ionizing radiation (radiologists, cardiologists and orthopaedic specialists) compared to 105 non-exposed controls [58]. The combination of macro- and micro-vascular damage can thus, in principle, exert negative effects on the neurovascular and neurocognitive function of subjects exposed to ionized radiation.

### Neurocognitive effects: direct radiation effects on neural cells

Terminally differentiated neurons have reduced or null proliferative capacity, so they have not been traditionally regarded as critical radiation targets. However, tissue tolerance of the normal brain to radiation therapy is very limited and radiation doses have to be tailored to minimize the deleterious effects on the nervous system [59]. Of special interest, cognitive decline in patients with radiological abnormalities (white matter hyperintensities and global cortical atrophy) was associated with radiotherapy doses that are considered safe (< 2 Sv) [60]. There are four possible cellular targets of radiation damage: endothelial cells, which are sensitive to radiation damage although they may recover after initial reduction in cell number [61]; oligodendroglial stem cells, which represent 75% of the cycling cells in the human brain and are permanently destroyed after high-dose radiation with subsequent delayed demyelinization [62]; microglial cells, which are mature cells that continue to divide in the CNS and are decreased in the spinal cord of irradiated rats [63]; neural stem cells giving rise to adult-born neurons [64]. At present there is consensus that adult neurogenesis occurs in two main areas (Figure 4) of the human brain: the subgranular zone (SGZ) of the dentate gyrus (DG) of the hippocampus, where new granule generated neurons have been associated with learning/memory and mood modulation, and the subventricular zone (SVZ), from which newborn cells migrate through the rostral migratory stream and give rise to newly generated neurons in the olfactory bulb [65,66]. Adult neural stem cells are highly sensitive to radiation even at chronic, moderate doses [67-70]. For example, with a cumulative dose of 0.5 Sv, in a mouse model, repetitive exposure more closely mirroring the model of professional exposure had a much more pronounced effect on cellular neurogenesis than acute exposure [70]. Reduction/arrest of adult neurogenesis in the hippocampus by low targeted X-irradiation impairs cognitive tests related to hippocampal memory [71,72]. Acute irradiation with sublethal dose causes reduction of cell proliferation and morphological alterations in the olfactory bulb with successive development of gliosis [73]. Interestingly, focal SVZ irradiation reduced the proliferation rate of newly generated olfactory neurons, thus resulting in long-term olfactory memory dysfunction; these data suggest that newborn adult neurons are involved in the memory of olfactory traces [74].

**Figure 5:**
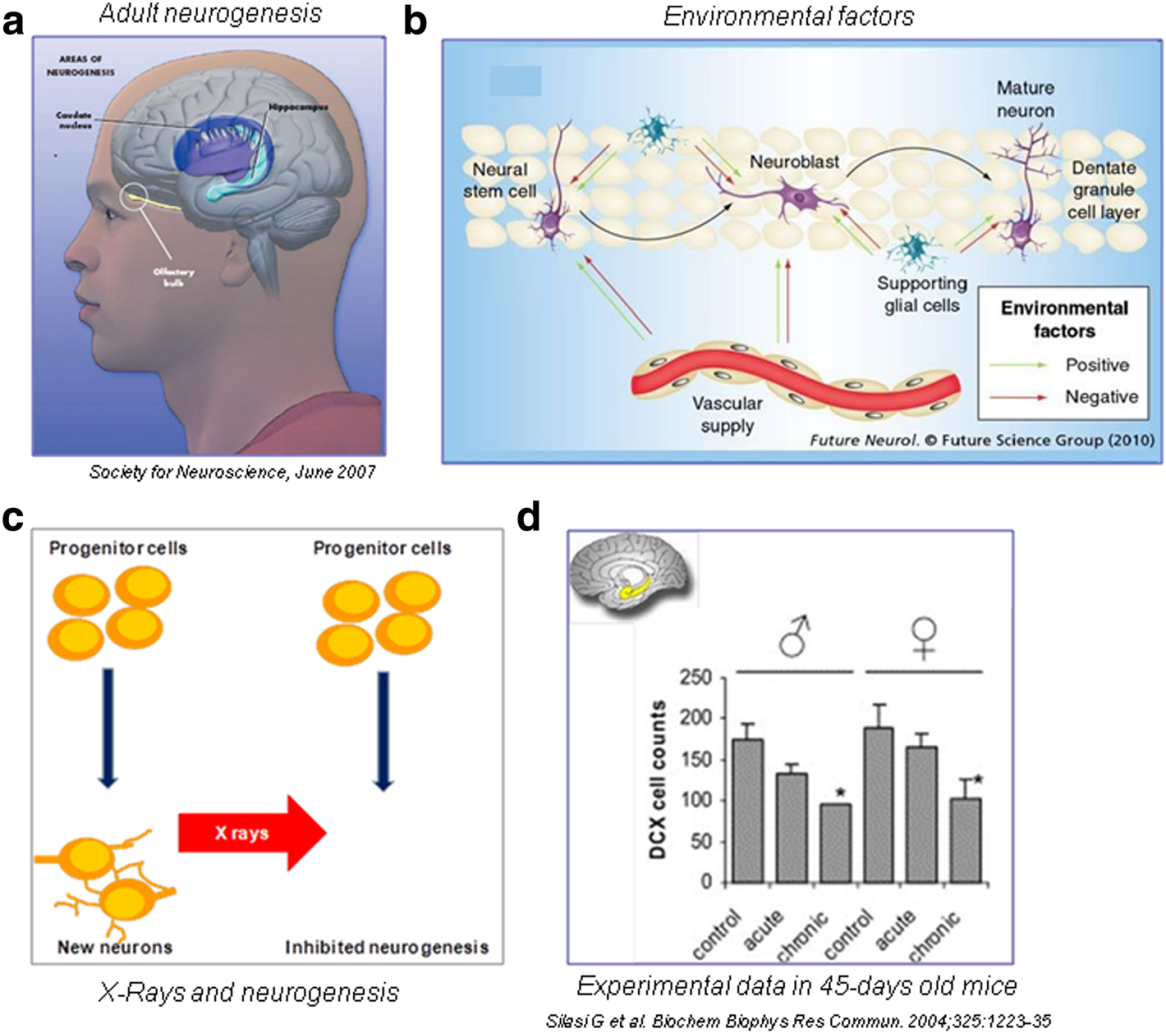
**Scatter dose rate values during fluoroscopy in interventional cardiologists without protection (as is was standard practice in many laboratories until some years ago).** The use of lead cap protection or total body protection with radioprotection cabin or ceiling suspended screen reduces the scatter dose to less than 1%. From the International Atomic Energy Agency collection of slides (Radiation Protection of Patients, on the dedicated and continuously updated website: http://rpop.iaea.org.website), ref 96 (Kuon 2003) and ref. 18 (Dragusin, 2007).

Previous studies suggested that radiation exposure might represent a risk for schizophrenia in humans [75,76]. In 10,834 individuals irradiated in childhood for tinea capitis (mean dose = 1.5 Gy), no association was found between radiation exposure and risk of schizophrenia, although for the subgroup irradiated at < 5 years of age a trend was found (hazard ratio = 1.18, 95% CI = 0.96–1.44, p = 0.1) [75]. Recent data showed that rats exposed to fractionated radiation dose present reduction of neurogenesis in DG and SVZ associated with schizophrenia-like behavior [77]. Finally, it should be remarked that neural stem cells and microglial cells can be impaired several years before clinically overt neurodegenerative diseases such as sporadic Alzheimer’s and Parkinson’s disease [66,78]. At a molecular level, the major fundamental mechanism triggered in the irradiated brain and responsible for structural alterations is DNA damage followed by pro-oxidant, pro-inflammatory and enhanced apoptotic response [79]. These effects have all been described in circulating lymphocytes and plasma of interventional cardiologists [80,81]. In the brain, apoptosis of neuronal stem cells and reduction of their proliferation rate following irradiation has been repeatedly associated with cognitive deficits in adult mammals [70-73]. However, the molecular substrate of cognitive impairment following low-dose radiation is still debated; in particular, the question of when and to what extent synaptic transmission/plasticity is affected by fractionated radiation is by and large unanswered. Previous results showed that hippocampal slices undergo changes in neuronal excitability following moderate doses of ionizing radiation [82]. More recently, in the mouse brain, Silasi et al. [70] reported perturbations in cell signaling associated with impairment of hippocampal neurogenesis. This issue is becoming even more relevant following recent observations by Mancuso et al. [25] on changes occurring in unexposed regions neighboring damaged cells, due to cell-to-cell communication or soluble factors released by irradiated cells. Thus, an accurate investigation of fractionated vs acute radiation damage to neuronal/neural cells in different brain areas is needed in order to understand the link between molecular mechanisms of radiation-induced alterations and cognitive impairment. This represents an important topic, since outside the field of radiation therapy [83], the evidence linking radiation exposure to cognitive disorders is weak, especially in the case of occupationally exposed medical workers [76,78]. Yamada et al. reported no relationship between radiation exposure (< 4 Gy) and dementia in 2,286 aging atomic bomb survivors [84]. Less reassuring data are available regarding occupational exposures in the low-to-moderate dose range (< 500 mSv). Death from dementia was significantly associated with total lifetime radiation doses in 69,976 female nuclear power plant workers [85], pre-senile dementia was more frequent in dentists [86], and elevated mortality from intentional self-harm, alcoholism and drowning was found in 11,311 former US flight attendants [87]. In 100 Chernobyl liquidators and 100 patients who suffered the acute radiation sickness in Chernobyl, schizophrenia-like disorders were more frequent in presence of over- irradiation (> 300 mSv) [88]. Mental disorders (including mental retardation and behavioral disorders) were most frequent in 544 Chernobyl prenatally irradiated children (with an estimated dose > 0.30 Sv to pregnant mothers) compared to non-irradiated controls born in radioecological “clear” regions [89]. There is no doubt that Chernobyl had an effect on mental health of adults directly affected by the event, especially the liquidators and women with young children, which is why the 2006 Chernobyl Forum report regarded mental health as the major public health consequence [90]. However, the scope and magnitude of the mental health effects cannot be specified with the data at hand [91]. The interpretation of these findings remains difficult due to confounding factors such as environmental mental stress, other possible chemical or physical contaminants in work habitat, night shift, and socio-economic confounders. Radiation is only a potential - but unproven - source of bioeffects, but certainly more data are warranted [92].

### Other non-cancer effects: cataract

The radiation protection standards formulated by the United States National Council on Radiation Protection and Measurements (NCRP) and the International Commission on Radiological Protection (ICRP) are all based on the belief that lens opacities (cataracts) are deterministic radiation-induced effects and appear only if a dose threshold is exceeded. Cataract, or opacification of the lens, is often associated with visual impairment and may be classified into three main categories: nuclear, cortical, and posterior subcapsular, according to their anatomic location [93]. Among the three major areas of age-related cataracts, posterior subcapsular is the least common but it is the one most frequently associated with ionizing radiation exposure. The mechanism of cataract formation remains partially unknown. There is a transparent layer of cells covering the interior frontal side of the capsule that covers the lens. This layer maintains the function of the lens by slowly growing toward the center, achieved through cell division at the periphery. Because radiation is especially harmful to dividing cells, exposed cells at the equator are most prone to damage. For unknown reasons, damaged cells move toward the rear of the lens before converging on the center. Such cells prevent light from travelling straightforward resulting in opacity. Because of their location along the lens’ visual axis, relatively minor posterior subcapsular cataracts can have great impact on vision. The estimated eye dose is around 0.5 mGy/procedure, when no eye protection is used. Until recently, the dose threshold for radiation-induced lens opacities were considered 2 Gy for a single dose or 5 Gy for fractionated dose [94]. However, several epidemiological studies among Chernobyl clean-up workers, A-bomb survivors, astronauts, residents of contaminated buildings, and surveys of staff in interventional rooms indicate that there is an increased incidence of lens opacities at doses below 0.5 Gy and even suggest a stochastic hypothesis (non-threshold effect) [95]. Whether deterministic or stochastic in nature, cataracts can be found in up to 50% of interventional cardiologists [96].

The reasons for this high prevalence are threefold: first is that operator’s eyes are exposed to scattered x-rays. Without lead protection, the operator’s eyes receive a mean entrance skin dose of 165 μSv per coronary angiography session, but the use of lead eyeglasses reduces this level to 37 μSv [96]. Second (avoidable) is the frequent failure of some cardiologists to use protective leaded eyewear [68]; and probably third, that the allowed occupational dose limits were too high to even keep an alert in mind. On April 21, 2011, ICRP slashed the earlier dose limit of 150 mSv in a year for the lens of the eye to the present 20 mSv in a year, averaged over a defined period of 5 years, with no single year exceeding 50 mSv [9].

### The unique model of interventional cardiologists and x-ray exposure: mind the brain!

**Figure 6:**
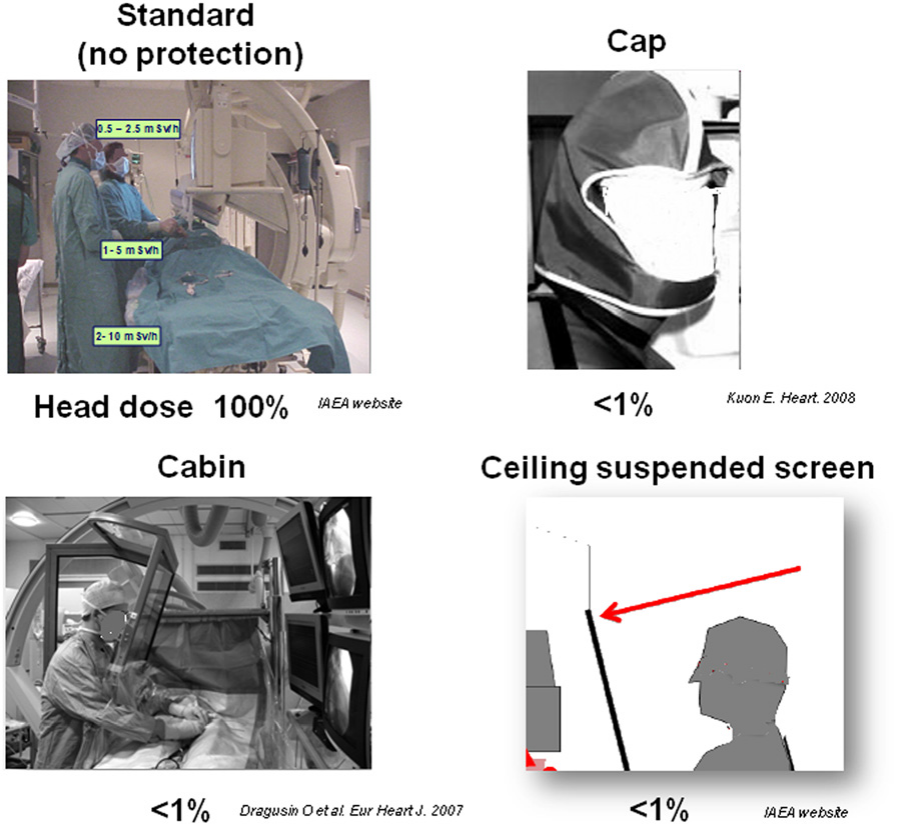
**Scatter dose rate values during fluoroscopy in interventional cardiologists without protection (as is was standard practice in many laboratories until some years ago).** The use of lead cap protection or total body protection with radioprotection cabin or ceiling suspended screen reduces the scatter dose to less than 1%. From the International Atomic Energy Agency collection of slides (Radiation Protection of Patients, on the dedicated and continuously updated website: http://rpop.iaea.org.website), ref 96 (Kuon 2003) and ref. 18 (Dragusin, 2007).

First-generation interventional cardiologists who entered the catheterization laboratory 20 to 30 years ago were inclined to believe that radiation is not such a serious matter [21], and in particular that the brain is a radio-resistant organ, that the eye lens can tolerate high doses of radiation, that no significant mitotic activity is present in the adult brain, and that no biologically plausible effect can link x-ray exposure to non-cancer disease such as neurovascular and neurodegenerative disorders. Unfortunately, all these assumptions have been disproved in the last decades. As a consequence, operators in catheterization laboratories should modify the habit of not using personal protective garments to shield their forehead and brain. The first line of whole-body defence against unwanted effects of radiation exposure is to start implementing the principles of justification and optimization in the catheterization lab. The second line of defence is to strictly adhere to protection practice (Figure 6). The use of a ceiling-suspended screen [21] or 0.5 mm lead cap attenuates scatter dose to the head by a factor of 2000 of baseline [96]. The radioprotection cabin reduces the head dose from over 100 μSv per procedure to the environmental background level of 1 μSv per procedure [18]. The third action is to start to look systematically to health (including brain) effects on the interventional cardiologist and staff population, which may become a Rosetta stone for decoding the long-term effects of radiation exposure, a recognized major environmental pollutant in the contemporary environment [97]. Contemporary invasive cardiologists and interventional radiologists are therefore a suitable research model for addressing many unanswered questions on the link between radiation exposure and proven but still imprecisely defined (eye cataract or brain cancer), probable (vascular) and possible (neuro-cognitive) serious health effects. In fact, two major studies on interventional cardiologists, invasive radiologists and catheterization laboratory staff are now at the starting blocks: the North American and the Italian studies (Table 3). The Multispecialty Occupational Health Group (MOHG) undertook a cohort mortality study comparing cancer and other serious disease outcomes (including cardiovascular disease and cataracts) in 44,000 physicians performing fluoroscopically guided procedures (including interventional cardiologists, radiologists, neuroradiologists and others) and in 12,000 non-interventional radiologists with risks in 101,000 physicians who are unlikely to be exposed to occupational radiation (e.g., family physicians or psychiatrists) [98]. Member organizations of the MSOHG include the Society of Cardiac Angiography and Intervention, Society of Interventional Radiology, Heart Rhythm Society, American College of Radiology, American College of Cardiology, Society of Neurointervention Surgery, American Association of Physicists in Medicine, and Society of Invasive Cardiac Professionals. The MSOHG is collaborating with experts in occupational health, epidemiology, and radiation effects who are from the United States Navy and the Radiation Epidemiology Branch of the National Cancer Institute, to perform epidemiological studies addressing the fundamental questions important to all those working in such an environment.

**Table 3:** Ongoing studies on interventional cardiologists

**Main funding**	**NIH and NCI**	**Italian CNR National Research Council-IFC, Institute of Clinical Physiology**
**Scientific Societies endorsement**	Multispecialty Occupational Health Group	Italian Society of Invasive Cardiology (GISE)
**Enrolled population**	· 44,000 fluoroscopists (interventional cardiologists, radiologists, neuroradiologists) · 42,000 non-interventional radiologists · 101,000 non-exposed physicians	· 500 exposed interventional cardiologists (nurses, technicians) · 500 non exposed clinical cardiologists (nurses, technicians)
**Endpoint**	Epidemiological clinical endpoints (cancer, cataract, vascular events)	Surrogate biomarkers of genetic, vascular, reproductive, cognitive effect

In Italy, The Healthy Cath Lab study is organized by the Italian National Research Council with endorsement of the Italian Society of Invasive Cardiologists, and is designed by interventional cardiologists for interventional cardiologists. The Italian study population will consist of 500 exposed (high, medium, and low exposure) interventional cardiologists and staff (technicians and staff) and 500 unexposed controls (clinical cardiologists and nurses). With this limited sample size, the detection of potentially increased health risks remains difficult using the epidemiological approach. Therefore, as an alternative to the epidemiological approach, the Healthy Cath Lab study will assess brain effects though “early warning signs”, which evaluate initial damage through surrogate endpoints that are easy to measure, non-invasive, and able to identify long-term risk for subsequent clinically overt disease. Other effects evaluated in the study are endocrine, reproductive, and atherosclerotic functions. Examples of surrogate end-points adopted in the study are carotid-intima media thickness for cerebrovascular atherosclerotic disease [99], olfactory dysfunction for neurodegenerative disorders [100], and circulating plasma brain-derived neurotrophin, which is directly linked to hippocampal neurogenesis and is reduced in pre-depressive and neurodegenerative conditions [101]. Both the North American and Italian studies will bring the safety issue center stage and are destined to increase awareness of ionizing radiation in the catheterization laboratory and generate relevant data for better understanding of the most serious health effects of professional chronic low-dose radiation exposure, eventually bridging the experimental and epidemiological divide between high-dose (radiotherapy) and chronic low- to moderate doses (professional exposure) [102]. Taken together, these studies should remind the interventional cardiology community that “the responsibility of all physicians is to minimize the radiation injury hazard to their patients, to their professional staff and to themselves” [103].

## **Conclusions**

The brain is among the most critical dose-limiting organs in radio-therapy, mainly due to the development of cognitive dysfunction following white matter disruption. The neuro-vascular unit is also vulnerable to radiation effects, and cerebro-vascular atherosclerotic damage is now considered proven with epidemiological evidences for doses > 500 mSv. The head doses involved in radiotherapy are high, usually above 2 Sv, whereas the low-dose range of professional exposure typically involves lifetime cumulative whole-body exposure in the low-dose range of < 200 mSv, but with head exposure which may (in absence of protection) arrive at a head equivalent dose of 1 to 3 Sv after a professional lifetime (corresponding to a brain equivalent dose around 500 mSv). At this point, a systematic assessment of brain (cancer and non-cancer) effects of chronic low-dose radiation exposure in interventional cardiologists and staff is needed.

## Abbreviations

CNS: Central nervous system; DG: Dentate gyrus; ICRP: International Commission of Radiological Protection; LDR: Low dose radiation; SGZ: Sub-granular zone; UNSCEAR: United Nations Scientific Committee on the Effects of Atomic Radiation.

## Competing interests

The authors declare that they have no competing interests.

## Authors’ contributions

EP is a clinical cardiologist and drafted the manuscript; all authors critically revised it and gave a critical intellectual contribution. In particular, EV is a medical physicist, who reviewed and contributed to writing the radiation exposure section; LD is a neurobiologist, who reviewed and contributed to writing the part on cognitive effects; MB is a statistician and epidemiologist, who reviewed and contributed to writing the part on brain cancer; ITC is a radiation physicist, who reviewed and contributed to writing the parts on radiation exposure and cancer. All authors read and approved the final version for submission.

## Pre-publication history

The pre-publication history for this paper can be accessed here:

http://www.biomedcentral.com/1471-2407/12/157/prepub
